# Radiation-Induced Alopecia after Endovascular Embolization under Fluoroscopy

**DOI:** 10.1155/2016/8202469

**Published:** 2016-12-15

**Authors:** Vipawee Ounsakul, Wimolsiri Iamsumang, Poonkiat Suchonwanit

**Affiliations:** Division of Dermatology, Faculty of Medicine, Ramathibodi Hospital, Mahidol University, Bangkok, Thailand

## Abstract

Radiation-induced alopecia after fluoroscopically guided procedures is becoming more common due to an increasing use of endovascular procedures. It is characterized by geometric shapes of nonscarring alopecia related to the area of radiation. We report a case of a 46-year-old man presenting with asymptomatic, sharply demarcated rectangular, nonscarring alopecic patch on the occipital scalp following cerebral angiography with fistula embolization under fluoroscopy. His presentations were compatible with radiation-induced alopecia. Herein, we also report a novel scalp dermoscopic finding of blue-grey dots in a target pattern around yellow dots and follicles, which we detected in the lesion of radiation-induced alopecia.

## 1. Introduction

Radiation-induced alopecia after fluoroscopically guided procedures, although infrequently reported in the dermatological literature, is possibly becoming more common due to an increasing use of endovascular procedures. It is characterized by geometric shapes of nonscarring alopecia confined to the area of radiation, usually asymptomatic without signs of scalp inflammation. We report a case of a 46-year-old man presenting with asymptomatic, sharply demarcated rectangular, nonscarring alopecic patch on the occipital scalp, 1 month after cerebral angiography with fistula embolization under fluoroscopy. His distinct clinical presentations together with a history of endovascular embolization under fluoroscopy were compatible with radiation-induced alopecia.

## 2. Case Report

**Figure 1:**
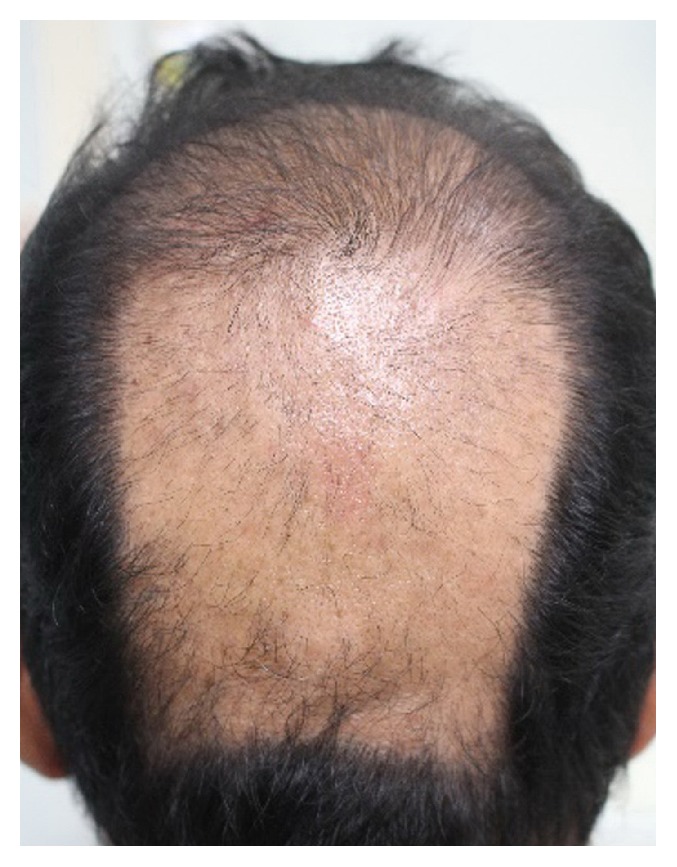
A sharply demarcated rectangular, nonscarring alopecia presented on the occipital scalp.

**Figure 2:**
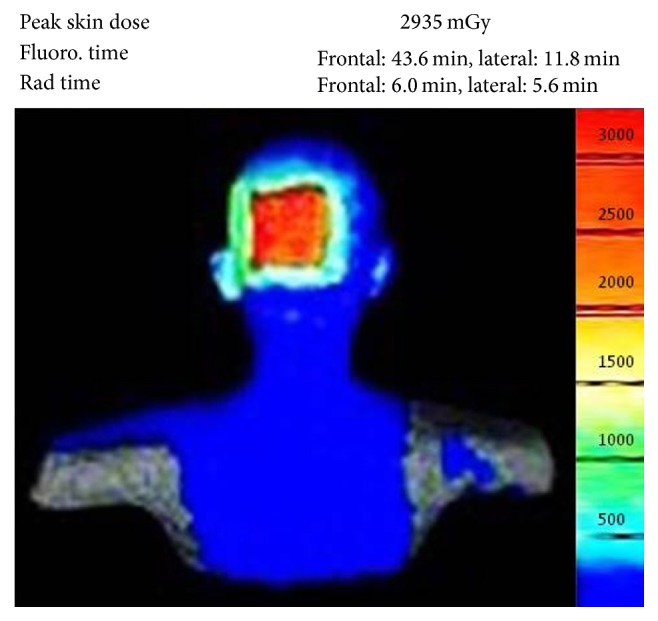
The rectangular shape of the radiation field in fluoroscopy-guided endovascular embolization is consistent with alopecic area in our patient.

**Figure 3:**
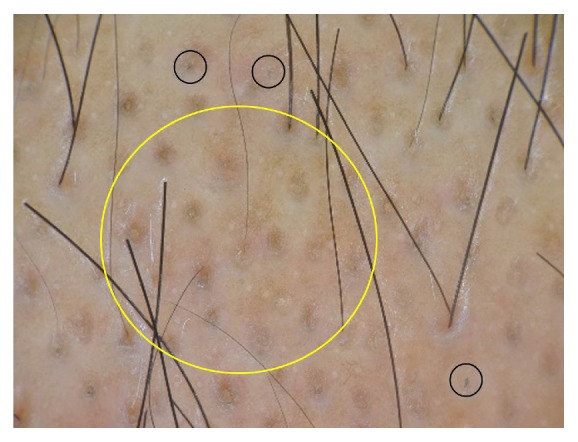
Dermoscopic examination showed mostly yellow dots (yellow circle), black dots (black circles), short vellus hairs, and blue-grey dots in a target pattern around yellow dots and follicles (polarized mode; 60x).

**Figure 4:**
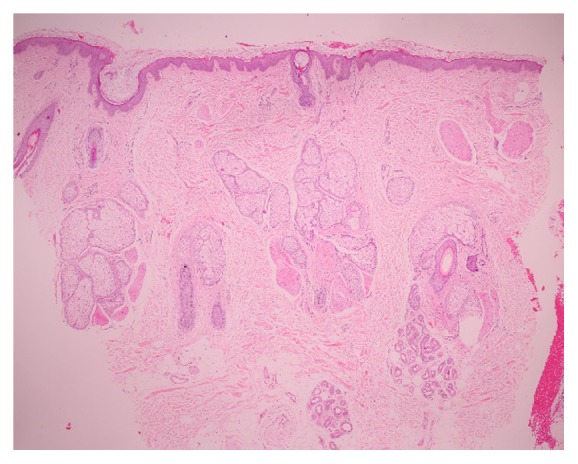
Histopathology showed increased numbers of catagen and telogen hairs, without perifollicular infiltration (hematoxylin and eosin staining; 20x).

**Figure 5:**
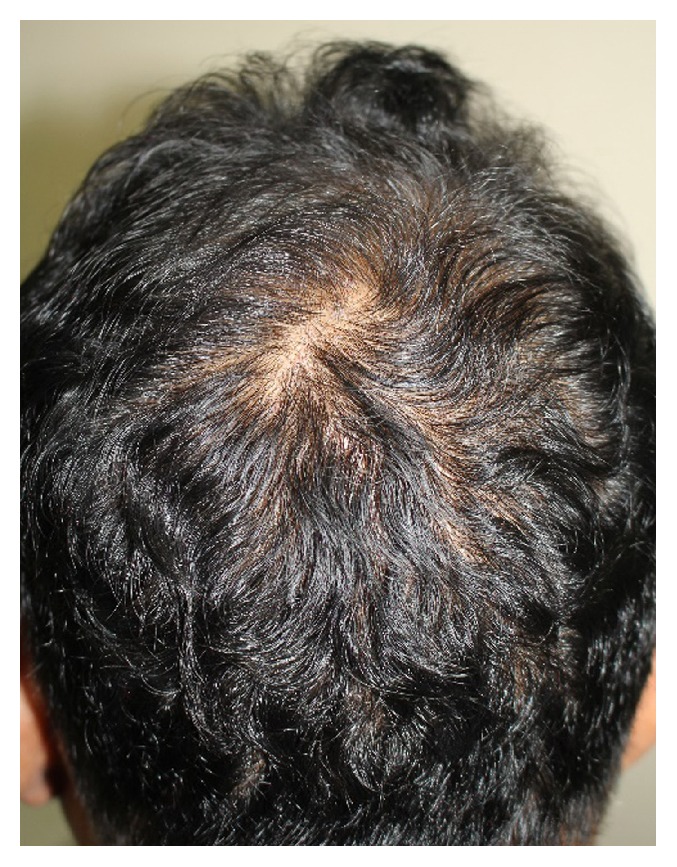
The patient had complete hair regrowth within 4 months.

A 46-year-old Thai man presented with asymptomatic sharply demarcated rectangular balding patch on the occipital scalp of 2 weeks' duration. Ten months earlier, he had a car accident and was diagnosed as having traumatic right carotid-cavernous fistula and optic neuropathy. Cerebral angiography revealed direct carotid-cavernous fistula from the C1 segment of cavernous part of the right internal carotid artery to the right cavernous sinus. Six months later, he was treated with transarterial balloon embolization without any complications. A month earlier, a follow-up magnetic resonance imaging and magnetic resonance angiography of the brain showed residual shunt. He underwent a cerebral angiogram with transvenous coil and glue embolization under fluoroscopy. The total procedure duration and the total radiation exposure time were 150 minutes and 67 minutes, respectively. The peak skin dose was 2.9 Gy. Two weeks after the procedure, he presented at the outpatient clinic with asymptomatic balding patch on his occipital scalp. Dermatologic examination revealed a sharply demarcated rectangular nonscarring alopecic patch, measuring 10 × 12 centimeters in size, on the occipital scalp, without erythema or scaling (Figures 1 and 2). Hair pull test was positive in the periphery of the alopecic patch and revealed 100% telogen hairs. Other physical examinations were unremarkable, except for the fact that visual acuity of the right eye showed only light perception. Dermoscopic examination of the alopecic area showed mostly yellow dots, black dots, short vellus hairs, and blue-grey dots in a target pattern around yellow dots and follicles (Figure 3). A 4 mm punch biopsy was performed and showed increased numbers of catagen and telogen hairs without perifollicular infiltration (Figure 4). Correlating the clinical presentation, history of radiation exposure, and compatible dermoscopic and histopathologic findings, the diagnosis of radiation-induced alopecia after endovascular embolization under fluoroscopy was established. We reassured the patient about the benign and self-limiting nature of the alopecia and also prescribed 5% minoxidil lotion to apply twice daily. The patient achieved complete hair regrowth within 4 months (Figure 5).

## 3. Discussion

Fluoroscopically guided endovascular procedures are becoming more common in medical practice with an increasing use of minimally invasive techniques for various medical conditions. In general, radiation-induced alopecia is common and develops following radiation therapy of neoplasms. However, alopecia secondary to radiation exposure from fluoroscopically guided procedures is increasingly being reported due to an increasing use of endovascular procedures.

Alopecia after fluoroscopically guided endovascular procedures is rarely reported in the dermatological literature, although this complication seems to be relatively common. Given that the procedures often require prolonged and repeated fluoroscopic imaging, doses above a deterministic threshold can potentially react with the skin and hair follicles [1, 2]. The exact incidence of the condition is still unknown. Jung et al. reported that 9 cases (6.7%) of 135 patients treated for aneurysm by coiling embolization suffered from alopecia [3].

Pathogenesis of radiation-induced alopecia involves acute damage to actively dividing matrix cells of anagen follicles causing immediate loss of dystrophic anagen hairs (anagen effluvium) and premature entry of some anagen hair follicles into catagen and then into telogen phase, resulting in delayed onset of hair shedding (telogen effluvium) [1]. Radiation threshold doses for deterministic effects of the skin proposed by the International Commission on Radiological Protection (ICRP) are 2 Gy for transient erythema, 3–6 Gy for temporary epilation, 7 Gy for permanent epilation, 10 Gy for moist desquamation, and 18 Gy for dermal necrosis [4, 5]. Our patient received radiation dose of 2.9 Gy which was slightly lower than temporary epilation range. Although the scalp is very resistant to radiation exposure, alopecia is paradoxically present at lower doses of radiation than the rest of the body. This is because actively dividing matrix cells in the anagen follicles have high susceptibilities to radiation [5, 6]. Severity of radiation-induced temporary alopecia depends on the dose, total duration, interval between each irradiation, size of area irradiated, angle of irradiation, and patient-related factors including smoking, poor nutritional status, preexisting autoimmune and connective tissue diseases, hyperthyroidism, and diabetes mellitus. Additionally, a great number of drugs have also been reported to increase radiosensitivity. These comprise actinomycin D, doxorubicin, bleomycin, 5-fluorouracil, methotrexate, mitoxantrone, cyclophosphamide, paclitaxel, docetaxel, and possibly tamoxifen [7–9].

Clinical presentation of radiation-induced alopecia includes geometric shapes of nonscarring alopecic patch confined to the area of radiation, usually asymptomatic without signs of scalp inflammation. Occipital, parietal, and temporal scalp are commonly affected. Occasionally, the alopecic area is clinically similar to other nonscarring alopecia cases such as alopecia areata. However, important clues to diagnosis are a history of fluoroscopically guided procedure and alopecic area which is confined to the radiation site. Hair loss mostly occurs within 1–3 weeks after radiation exposure with spontaneous regrowth of hair within 2–4 months [10–14]. Cho et al. reported 10 patients developing alopecia after angioembolization with mean duration of 3.4 weeks, ranging from 1 to 8 weeks, after procedure. All patients had a rectangular-shaped, alopecic patch on the occipital and temporal area, and 9 of 10 patients had complete hair regrowth within 3-4 months [15]. Our patient also presented with rectangular alopecia on the occipital scalp at 4 weeks after radiation exposure and achieved complete hair regrowth within 4 months aided with the use of twice-daily 5% minoxidil application.

Diagnosis of radiation-induced alopecia is primarily based on distinct clinical presentations and history of radiation exposure. Investigations including trichogram, dermoscopy, and histopathology are more likely to exclude other causes of nonscarring alopecia rather than confirming the diagnosis. Trichogram performed during an early phase usually shows dystrophic anagen hairs. However, in our patient, the presence of telogen hairs without dystrophic anagen in trichogram was a result of prolonged time from radiation exposure to the examination. All dystrophic anagen hairs shed off in the first few weeks after radiation exposure. A study of 10 patients with postangioembolization alopecia by Cho et al. reported that dermoscopic findings demonstrated both yellow dots and black dots (60%), short vellus hair (50%), peripilar sign (20%), broken hair (10%), coiled hair (10%), and white dots (10%), whereas histopathology showed increased numbers of catagen and telogen hairs without peribulbar inflammatory cell infiltrate, unlike alopecia areata, which usually shows peribulbar inflammation [15]. Although the dermoscopic findings of radiation-induced alopecia are nonspecific and share some common features with alopecia areata, the presence of peribulbar inflammation in histopathologic examination, which is commonly observed in alopecia areata, can help distinguish between these two conditions. In our patient, dermoscopic examination revealed mostly yellow dots, black dots, short vellus hairs, and blue-grey dots in a target pattern around yellow dots and follicles. To the best of our knowledge, the finding of blue-grey dots in radiation-induced alopecia has not yet been reported in the literature. Furthermore, the blue-grey dots detected in our case differ from those described in lichen planopilaris in which the blue-grey circular arrangements locate around yellow dots and follicles, rather than around white dots and follicles as described in lichen planopilaris [16]. The histopathologic examination showed increased numbers of catagen and telogen hairs without perifollicular infiltration. In the same way as in trichogram, there was an increase in numbers of catagen and telogen hairs, which was the finding in late stage, whereas dystrophic anagen hairs were not found because of their early shedding. We hypothesize that the presence of blue-grey dots in a target pattern may be due to hyperpigmentation following radiation. However, the corresponding histopathologic features such as the presence of melanophages or pigmentary incontinence were not found in our patient's section. This could be assumed by the fact that the area examined by dermoscopy and the area which underwent biopsy were not perfectly matched.

Other possible mechanisms of alopecia after fluoroscopy-guided endovascular embolization including alopecia secondary to an impairment of the external carotid blood supply of the scalp and pressure-induced alopecia should be also considered. The impairment of the external carotid blood supply of the scalp may result from therapeutic embolization of blood vessels or radiation-induced luminal fibrosis [1]. Owing to the patient's optimal superficial temporal and occipital arterial pulses, alopecia due to inadequate blood supply is unlikely. Additionally, pressure-induced alopecia, which has been reported to occur after prolonged operation, immobilizing illness, or direct blunt trauma, should be taken into account because of the rectangular shape of the alopecic patch. However, patients with pressure-induced alopecia typically report tenderness, swelling, or crusting prior to alopecia [17, 18]. Given that the posteroanterior view of the fluoroscopy as well as the rectangular shape of radiation field are consistent with asymptomatic well-demarcated hair loss on the occipital area in our patient, the diagnosis of radiation-induced alopecia was confidentially confirmed.

Treatment of radiation-induced alopecia is usually unnecessary due to its benign and self-limiting nature. Complete hair regrowth generally occurs within 2–4 months after irradiation [10–14]. In addition, no treatment or preventive measures appear to be generally effective [19, 20]. To prevent this unwanted effect, awareness and limitation of radiation exposure to the patient are the most important. Although topical application of epinephrine or norepinephrine before radiation was found to confer up to 95% coat retention in a rat model, further studies in human are required to indicate its efficacy [19]. Online monitoring of the patient's radiation exposure is also necessary because fluoroscopy time does not account for either fluoroscopic dose rates or the use of fluorographic acquisition modes during a procedure. However, in our case, twice-daily use of minoxidil 5% was administered due to the patient's need of treatment and its positive effects on hair growth.

In conclusion, we report the case of radiation-induced temporary alopecia after endovascular embolization under fluoroscopy. In addition to other nonscarring alopecia cases, physicians should be aware of and include this condition in the differential diagnosis when individuals presenting with nonscarring alopecia had a history of prolonged fluoroscopic endovascular procedures. Although the condition is benign and self-limiting, awareness, monitoring, and limitation of radiation exposure to the patient are the most important strategies to prevent hair loss.
